# Neural and vascular contributions to sensory impairments in a human alpha-synuclein transgenic mouse model of Parkinson’s disease

**DOI:** 10.1177/0271678X251338952

**Published:** 2025-05-07

**Authors:** Ruxanda Lungu, Francisca F Fernandes, Sara Pires Monteiro, Tiago F Outeiro, Noam Shemesh

**Affiliations:** 1 Champalimaud Research, Champalimaud Foundation, Lisbon, Portugal; 2 Institute for Systems and Robotics - Lisboa and Department of Bioengineering, Instituto Superior Técnico – Universidade de Lisboa, Lisbon, Portugal; 3 Department of Experimental Neurodegeneration, University Medical Center Göttingen, Center for Biostructural Imaging of Neurodegeneration, Göttingen, Germany; 4 Max Planck Institute for Multidisciplinary Sciences, Göttingen, Germany; 5 Translational and Clinical Research Institute, Faculty of Medical Sciences, Newcastle University, Newcastle Upon Tyne, UK; 6 Scientific Employee with an Honorary Contract at German Center for Neurodegenerative Diseases (DZNE), Göttingen, Germany

**Keywords:** Parkinson’s disease, *α*-Synuclein, fMRI, ASL, c-FOS

## Abstract

Parkinson's disease (PD) is a complex progressive neurodegenerative disorder involving hallmarks such as α -Synuclein ( α Syn) aggregation and dopaminergic dysfunction that affect brain-wide neural activity. Although movement disorders are prominent in PD, sensory impairments also occur relatively early on, mainly in olfactory and, to a lesser extent visual systems. While these deficits have been described mainly at the behavioral and molecular levels, the underlying network-level activity remains poorly understood. Here, we harnessed a human α Syn transgenic mouse model of PD with *in vivo* functional MRI (fMRI) to map evoked activity in the visual and olfactory pathways, along with pseudo-Continuous Arterial Spin Labeling (pCASL) and c-FOS measurements to disentangle vascular from neuronal effects. Upon stimulation with either odors or flickering lights, we found significant decreases in fMRI responses along both olfactory and visual pathways, in multiple cortical and subcortical sensory areas. Average Cerebral Blood Flow rates were decreased by ∼10% in the α Syn group, while c-FOS levels were reduced by over 50%, suggesting a strong neural driver for the dysfunction, along with more modest vascular contributions. Our study provides insight into brain-level activity in an α Syn-based model, and suggests a novel target for biomarking via quantification of simple sensory evoked responses.

## Introduction

Parkinson’s disease (PD) is the second most common neurodegenerative disorder,^1^ typically characterized by severe dopaminergic dysfunction, motor deficits, cognitive decline, and ultimately by severe disability. The etiology of PD is complex, involving an interplay between genetic, molecular, and environmental factors.^2^ In PD, α -Synuclein ( α Syn) aggregates in inclusions known as Lewy bodies and Lewy neurites, that spread through different brain regions with disease progression.^3,4^ Progressive changes in cellular and white matter microstructure have also been reported to link with cognitive decline.^5^ Brain atrophy, including volumetric decreases in dopaminergic areas,^6^ cortical thinning,^7^ and gray matter loss,^7^ also typically occur. Deficits in neural activity,^8^ both in dopaminergic circuits^9^ (e.g. basal ganglia and substantia nigra), and in more global networks (default mode network,^10^ sensorimotor network^11^ and cognitive control network^12^) have been described both in clinical and in preclinical^13,14^ settings. These factors contribute to PD’s hallmark motor symptoms, such as akinesia, bradykinesia, tremor and rigidity, gait disturbance, grip force and speech deficits, among others.^15^

Apart from these motor-related effects, PD also involves considerable sensory deficiencies. A reduced or lost sense of smell^16^ was proposed to start manifesting early – even years before clinical motor signs.^17^ To a lesser extent, visual impairments have also been described,^18^ including visual hallucinations, decrease in visual acuity, eye tremor movement, or visual processing speeds.^19^ Neuroimaging studies have found decreased metabolic consumption in some sensory areas,^20^ but whether these are due to decreased activity or due to e.g. brain atrophy or blood flow decreases still remain to be elucidated. Interestingly, animal models of PD,^21^ which allow for investigating the underlying mechanisms more directly and have mainly focused on motor and dopaminergic effects, have been able to recapitulate visual^22^ and (separately) olfactory^23^ deficits, mainly behaviorally.^23^ In a toxic PD rodent model^24^ where predominantly dopaminergic areas are injured, neural activity in visual areas such as Superior Colliculus were found to be enhanced upon visual stimulation,^25^ while in a local expression model of α Syn in the substantia nigra, the latency of visual evoked potentials (VEPs) in the SC was found to be decreased.^26^ Olfactory deficits have been linked with aberrant projection patterns,^27^ local perturbations of synaptic transmission in the bulb,^28^ reduced neurogenesis,^29^ and microglial activation.^30^ How these local effects affect neural activity in the entire networks, however, is less well understood.

*α*Syn transgenic models are playing an increasingly important role for understanding PD and sensory/motor effects. For instance, Zhang et al. (2015)^31^ reported deficits in odor discrimination and detection in Prp-A53T-*α*Syn mice at 6 months old. Similarly, Petit et al. (2013)^32^ reported age-dependent olfactory deficits in F28 mice overexpressing human wildtype *α*Syn. The *α*Syn BAC transgenic mice exhibited olfactory bulb pathology and related behavioral deficits by 5 months of age,^33^ underscoring that olfactory dysfunction typically emerges during the early stages of disease progression in these models. Visual impairments in αSyn transgenic mouse models were also noted, for instance with the overexpression of human wild-type α-Synuclein in the retina which has been shown to lead to early loss of dopaminergic amacrine cells, resulting in decreased visual acuity and altered electroretinography responses.^34^ Similarly, the Plp-α-Syn mouse model exhibits human α-Synuclein accumulation in retinal neurons, particularly rod bipolar cells, leading to mild functional alterations under low-light conditions.^35^ Furthermore, A53T αSyn overexpressing mice demonstrate reduced light-adapted ERG responses and outer retinal thinning correlating with elevated α-Synuclein levels.^36^ These findings suggest that visual deficits can manifest in αSyn transgenic models, potentially preceding or coinciding with motor symptoms. On the other hand, motor symptoms show greater variability depending on the specific *α*Syn transgenic model. In A53T *α*Syn mice, motor deficits such as bradykinesia and ataxia appear around 8–10 months of age, with progressive worsening.^37^ In contrast, other models, such as Line 61 mice, exhibit severe motor impairments as early as 1 month of age.^38^ These differences highlight the importance of model-specific considerations when interpreting motor dysfunction.

Functional MRI (fMRI)^39,40^ interrogates brain-wide activity in small rodents,^41^ completely noninvasively and with good spatiotemporal resolution, leading to deep insights into network-level function.^40,42^ In lesion-based models of PD, resting-state fMRI has shown increased functional connectivity in nigrostriatal pathways, which also extended to sensory cortices,^43–45^ suggesting higher baseline stimulation.^46^ In an α Syn-injection model, task-based fMRI found unchanged visual responses compared to controls.^47^ In addition, global cerebral blood flow (CBF) changes were found in a genetic α Syn model^48^ (as well as in a different MitoPark model^49^) making the results even more difficult to interpret at the network level, given the reliance of conventional fMRI on complex neurovascular coupling mechanisms,^50^ and the involvement of α Syn in vascular impairments.^51^

Given the importance of α Syn in PD and the questions of network-level sensory brain activity, we harnessed a well-established human α Syn transgenic (tg) mouse model at 9 months of age (early-to-mid disease progression^52^) along with task-based fMRI for a brain-wide, network-level perspective of neural activity, while disentangling potential neurovascular coupling ambiguities through c-FOS^53^ and CBF measurements.^54,55^ This α Syn mouse model has been extensively validated and mirrors important aspects of sporadic PD at the molecular^56^ and cellular levels,^57^ including the accumulation of α Syn, mitochondrial dysfunction,^58^ and neuroinflammation.^59^ Furthermore, this model consistently exhibits dopaminergic neuron loss in the substantia nigra,^60^ a hallmark of PD, and exhibits behavioral symptoms including bradykinesia, rigidity, postural instability, and tremors^56,61^ as well as non-motor symptoms such as gastrointestinal and anxiety-like behaviors.^58^ We hypothesized that given its genetic α Syn background, sensory processing deficits at the network level would be found in this mouse model. At this stage, olfactory and visual impairments are typically well-established,^52^ while motor deficits may begin to emerge.^38^ To avoid the confounding effects of neurovascular coupling (or decoupling^62^) we harnessed c-FOS expression measurements – a technique relying on immediate early gene expression^53^ – to examine the extent of underlying neuronal activity in critical junctions of the olfactory and visual pathways. We further employed pseudo-continuous Arterial Spin Labeling (pCASL) MRI^63^ for quantitative CBF mapping, to measure flow in the vascular compartment. Our findings reveal decreased fMRI signals upon both olfactory and visual sensory networks, explained mainly by reduced neural activity as well as globally decreased CBF, suggesting a more global sensory failure at the network level in these mice.

## Subjects and methods

All animal experiments were pre-approved by the competent institutional and national authorities , namely, the Champalimaud Animal Welfare Body and the Portuguese Direção-Geral de Alimentação e Veterinária, and were carried out according to European Directive 2010/63 and ARRIVE guidelines.

### Mouse line

The α Syn transgenic mouse model (C57BL/6-DBA/2 Thy1- α Syn) was used in this study.^60^ Transgenic mice ( α Syn; *N = *42) and their wildtype littermates (healthy controls (HC); *N = *43) were housed in a temperature-controlled room and kept in a 12 h/12 h light/dark cycle with *ad libitum* access to food and water. Of these animals, *N = *12 α Syn (male, 32.1 ± 6.7 g, 8.8 ± 0.9 months old) and *N = *13 HC (male, 47.6 ± 9.7 g, 8.6 ± 0.9 months old) underwent visual fMRI experiments, *N = *10 α Syn (male, 33.2 ± 3.3 g, 8.6 ± 0.7 months old) and *N = *10 HC (2 females, 38.9 ± 7.9 g, 9.1 ± 0.7 months old) underwent olfactory fMRI experiments, *N = *6 α Syn (male, 37.0 ± 6.2 g, 9.0 ± 0.4 months old) and *N = *6 HC (male, 37 ± 6 g, 9.0 ± 0.2 months old) underwent visual c-FOS detection experiments, *N = *6 α Syn (male, 38.0 ± 5.9 g, 9.0 ± 0.3 months old) and *N = *6 HC (male, 38 ± 6 g, 9.0 ± 0.2 months old) were used for olfactory c-FOS detection experiments, and *N = *8 α Syn (male, 37 ± 4 g, 9 months old) and *N = *8 HC (male, 37 ± 4 g, 9 months old) underwent pCASL experiments for measuring CBF (animal weights and ages are reported as mean ± SD). All animals were randomly chosen from a cohort of α Syn and HC mice.

### MRI setup

All MRI scans were performed on a 9.4 T Bruker BioSpec scanner (Bruker BioSpin, Karlsruhe, Germany) equipped with an 86 mm volume coil for transmittance and a 4-element array cryogenic coil (Bruker BioSpin, Fallanden, Switzerland) for reception, a gradient system capable of producing up to 660 mT/m isotropically, and running ParaVision 6.0.1 software (Bruker Biospin, Ettlingen, Germany).

### fMRI experiments

#### Animal preparation

**Figure 1.**
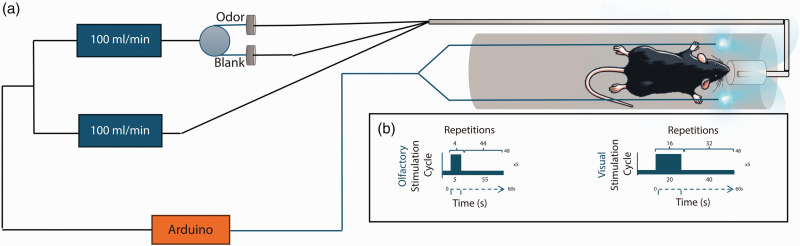
Experimental setup for fMRI experiments. (a) The odor delivery setup uses an olfactometer to deliver odors directly to the mouse nose. Two blue LEDs placed on each side of the head deliver binocular visual stimuli and (b) The fMRI paradigms used for each type of stimulation. The systems are controlled via an Arduino controller and are TTL’ed by the MRI scanner to ensure correct presentation of stimuli.

Anesthesia was induced with 5% isoflurane (Vetflurane®, Virbac, France) mixed with oxygen-enriched (28%) medical air. Mice were weighed, moved to an MRI compatible bed (Bruker Biospin, Germany) equipped with odor/visual stimulation capacity (Figure 1) and isoflurane was reduced to 2–3%. The mouse head was secured with ear and bite bars, and eye drops (Bepanthen® Eye Drops, Bayer AG, Germany) were applied. About 6 min after isoflurane induction, animals were sedated with medetomidine s.c. (0.4 mg/kg initial bolus and 0.8 mg/kg/h continuous infusion). Isoflurane was gradually discontinued along the first 15 min of the experiment, allowing a smooth transition between isoflurane anesthesia and medetomidine sedation.

Rectal temperature and respiration rate were continuously monitored (SA Instruments, Inc., USA) and remained stable throughout the sessions. A warm water recirculating pad was used for body temperature control. At the end of experiments, an s.c. injection of 2 mg/kg atipamezole was administered to all animals to reverse medetomidine effects. All animals visibly recovered in ∼10 min.

#### Visual stimulation

The two ends of a two-branching optic fiber coupled to a blue LED (λ = 470 nm) were placed at ∼5 mm from the animals’ eyes for binocular stimulation flashing at 1 Hz frequency with a pulse width of 10 ms.^64^ The fMRI design consisted of five blocks of 40/20 s of rest/stimulation, respectively, followed by a rest period of 40 s, and was repeated 1–3 times per animal.

#### Olfactory stimulation

Mice were stimulated using a specialized odor delivery setup^65^ (Figure 1) providing Amyl acetate diluted 1:10 in mineral oil for odor stimulation. The fMRI paradigm was composed of five blocks of 55 s of rest followed by 5 s of continuous olfactory stimulation, ending with 55 s of rest, and was repeated 1–7 times per animal.

#### MRI acquisitions

After initial adjustments and localizer scans, two T_2_-weighted Turbo RARE sequences were acquired in the coronal and sagittal planes for accurate anatomical referencing (TR/TE = 2500/33 ms, FOV =25 × 25 mm^2^ (coronal) or 20 × 20 mm^2^ (sagittal), in-plane resolution = 98 × 98 µm^2^, RARE factor = 8, slice thickness = 0.7 mm, slice gap = 0.3 mm, number of slices = 14 (coronal) or 11 (sagittal), t_acq_=2 min 40 s (coronal) or 2 min 5 s (sagittal)).

All fMRI acquisitions began at least 40 min after isoflurane induction to ensure sufficient anesthetic washout. A multislice GE-EPI sequence with the following parameters was used for fMRI scans: for visual stimulation experiments, TR/TE = 1250/12 ms, flip angle = 75°, FOV = 15 × 12 mm^2^, in-plane resolution = 150 × 150 μm^2^, partial Fourier factor in the PE direction = 0.77 (acceleration factor = 1.3), slice thickness = 0.45 mm, slice gap = 0.1 mm, number of slices = 14 slices, repetitions = 272, t_acq_ = 5 min 40 s, dummy scans = 216 (to ensure gradient temperature stabilization); and for olfactory stimulation experiments, TR/TE = 1250/12 ms, flip angle = 75°, FOV =16 × 12 mm^2^, in-plane resolution = 200 × 200 μm^2^, partial Fourier factor in the PE direction = 0.77, slice thickness = 0.45 mm, slice gap = 0.1 mm, number of slices = 10 slices, repetitions = 284, t_acq_ = 5 min 55 s, dummy scans = 172.

#### Data analysis

All datasets were analyzed in MATLAB® (MathWorks, USA) using custom written code calling SPM12 functions. Functional data were first outlier corrected slicewise by manually selecting time points whose average brain signal deviated ∼2–3 SD from its 2^nd^ order polynomial trend, and estimating new voxel values at those time points using piecewise cubic interpolation from the signal at the adjacent time points. Only <0.08% of datapoints/scan were identified as outliers and corrected. Images were then slice-timing and motion corrected, coregistered to their respective coronal anatomical scan, and normalized to the Allen Reference Atlas^66^ in SPM12.

#### Voxelwise GLM analysis

Brain Oxygen Level Dependent signal (BOLD) mapping was analyzed in SPM12, using a general linear model (GLM) approach. Data were first smoothed with a 3 D isotropic Gaussian kernel (FWHM = 150 μm if visual fMRI or 200 μm if olfactory fMRI in all directions). For both visual and olfactory fMRI data, the experimental regressor of the design matrix was obtained by convolution of the respective stimulation paradigm with a double gamma HRF so that the expected BOLD response would peak at 3 s after stimulus onset. Motion correction parameters were used as nuisance regressors. For visual fMRI data only, the global brain signal was used as an extra nuisance regressor^67^ due to brain-wide fluctuations in some scans. A high-pass filter with a 120 s cutoff was incorporated into the GLM to correct for slow signal drifts. A fixed-effects group analysis was run independently for each group and for the difference between both groups. Resulting *t*-value maps were thresholded with *p* < 0.001 (when analyzing each group) or *p* < 0.01 (when analyzing the difference between groups) and a minimum cluster size of 10 voxels and were cluster-FDR corrected at *p* < 0.01.

#### ROI analysis

For visual fMRI data, signals were extracted from four anatomical bilateral ROIs obtained from the Allen Reference Atlas^68^ corresponding to different visual pathway structures, namely the superior colliculus (SC), primary visual cortex (V1) and dorsal part of the lateral geniculate nucleus (LGN), and to a control region, the dentate gyrus (DG); data were then averaged for each ROI. For olfactory fMRI data, signals were also extracted and averaged inside the ROI for some olfactory pathway structures like the piriform cortex (PIR) and for a control region, the primary motor cortex (MOp). The main olfactory bulb (MOB) was also divided into three different layers that approximately covered, respectively, the glomerular layer (GL), the external plexiform, mitral cell and internal plexiform layers (EPL_MCL_IPL), and the more internal granular cell layer (GCL). These were manually defined according to the Paxinos and Franklin’s atlas.^69^ Each ROI time-course was then subjected to global signal regression (visual fMRI data only), detrended using a 2^nd^ degree polynomial fit to the first resting period and last 10 s of the remaining resting periods to remove low frequency trends and converted to percentage signal change. Responses were then averaged across all animals in each group to obtain the average full time-course, or across all stimulation epochs and animals to obtain the average cycle, from which mean ± SD values were extracted and the 95% confidence interval (CI) calculated.

### c-FOS experiments

#### Measurement of c-FOS levels

Mice were kept in darkness/silence for 24 h before the experiment and were prepared in the same way as fMRI experiments (i.e., with the same induction and sedation protocol) while still in darkness/silence. They were then placed in a mock MRI bed to mimic the scanner setup and were then exposed to identical visual or olfactory stimulation paradigms as during the fMRI experiments (5 cycles per animal). Fixation was performed at 90 min as recommended,^70^ while the animal remained in darkness and under continuous medetomidine infusion.

#### Brain extraction and sample preparation for immunohistochemistry

Brain specimens were fixed via standard transcardial perfusion. Briefly, animals were injected i.p. with an overdose of Pentobarbital and the entire body and brain were perfused.^71^ The brain was extracted from the skull, immersed in a 4% PFA solution for 24 h and washed in a PBS solution thereafter, for immediate brain slicing. Brain slices for microscopy were obtained using a Vibratome (Leica VT1000s, Germany) sectioning with a thickness of 0.05 mm in 10 different horizontal levels spanning the entire olfactory and visual regions.

The samples were then washed in PBS 1x concentrated and incubated in PBS 10%/Triton 0.3 (PBST) for 1 h. Subsequently, the slices were incubated with primary antibodies (Rabbit monoclonal recombinant IgG anti c-FOS) at a 1:4000 dilution in 10% bovine serum albumin (BSA) + 0.1% sodium azide + 0.3% PBST overnight. The samples were then washed again three times with PBS and incubated in 10% BSA in 0.3% PBST for 2 h, then incubated with the secondary antibodies (Goat Anti-Rabbit IgG H&L (Alexa Fluor® 488)) at a 1:1000 dilution in 10% BSA + 0.1% sodium azide + 0.3% PBST, for 2 h. Finally, the samples were washed in phosphate-buffer (PB) 0.1 M at least three times, and then mounted in SuperFrost slides and 120 μl of Mowiol containing 2.5% 1,4 diazobicyclo [2.2.2]-octane (DABCO, Sigma, D2522) and high resolution (#1.5) coverslips were added.

#### Microscopy and cell counting

Microscopy was performed using a ZEISS Axio Scan.Z1 (Zeiss, Germany) coupled to a CCD color camera (Hitachi 3, Oxford instruments, UK). Images were then processed with Stereo Investigator® (MBF Bioscience - VT, USA).^72^ For animals that underwent visual stimulation, ROIs were selected in V1, SC, LGN, and the entorhinal cortex (EC) as a control area. For animals that underwent olfactory stimulation, ROIs were selected in the GL, EPL_MCL_IPL and GCL of the MOB, and EC served as a control area. Expression levels were extracted from these regions in both groups. Statistical analyses using a Shapiro-Wilk test were performed at a 5% significance level, confirming the normality of the data distributions. Consequently, *t*-tests were used to compare expression levels between groups. Cohen’s *d* measurements of effect size were also calculated, using Sawilowsky’s expansion for interpretation of these values.

### ASL experiments

To assess perfusion differences between α Syn and HC, two new groups of animals underwent pCASL perfusion experiments 48 hours after being subjected to an fMRI session. Animals were anesthetized using a mixture of medical air and 3.5% isoflurane (Vetflurane®, Virbac, France) and were maintained at 1.5–2.5% throughout the experiment. Mice were positioned on top of a custom designed ramp for proper carotid positioning as recently described by Pires Monteiro et al. (2024).^73^ An unbalanced pCASL sequence was used,^74^ and the labeling plane was positioned at the mouse neck (∼8 mm below the isocenter). pCASL acquisitions were achieved with the following parameters: single-shot EPI, Bandwidth = 300 kHz, LD (labeling duration) = 3 s, PLD (post labeling delay)=300 ms, FOV = 12 × 12 mm^2^, an in-plane spatial resolution of 100 × 100 μm^2^, slice thickness = 0.5 mm, slice gap =0.35 mm, TR/TE = 4000/25 ms, 30 control-label repetitions, t_acq_ = 4 min.

#### CBF quantification

For CBF quantification, a T_1_ map was obtained from an inversion recovery spin-echo EPI sequence (TR/TE = 10000/19 ms; 18 inversion times (TI) between 30 and 10000 ms; t_acq_ = 4 min). In addition, a pCASL encoded fast low angle shot (FLASH) sequence was used to estimate the inversion efficiency by acquiring the signal 3 mm above the labelling plane (TR/TE = 225/5.6 ms; slice thickness =1 mm, 2 averages, PLD = 0 ms, LD = 200 ms, t_acq_ = 3 min 30 s). CBF maps (ml/100g/min)^75^ were calculated pixel-by-pixel via equation (1):

(1) $$\mathrm{CBF}=\frac{6000\times \lambda \times \Delta M\times e^{\frac{\mathrm{PLD}}{T_{1}^{b}}}}{2\times \alpha \times T_{1}^{t}\times M_{0}^{t}\times {\left(1-e^{-\frac{TR}{T_{1}^{t}}}\right)}^{-1}\times \left(1-e^{\frac{-LD}{T_{1}^{t}}}\right)}$$

where $T_{1}^{b}$ and $T_{1}^{t}$ are the longitudinal relaxation times of blood (assumed to be 2430 ms at 9.4 T^76^) and tissue (extracted from the T1 map in each voxel), respectively. ΔM is the difference in magnitude between control and label images, averaged across the 30 repetitions to ensure sufficiently large robustness in CBF mapping. The equilibrium magnetization of blood can be approximated by $\frac{M_{0}^{t}}{\lambda }$ , where $M_{0}^{t}$ is the tissue magnetization in the average control image and λ is the blood-tissue partition coefficient of water (taken as the brain average 0.9 mL/g^77^).

## Results

### Disrupted BOLD-fMRI signals in the olfactory pathway of α Syn mice

**Figure 2.**
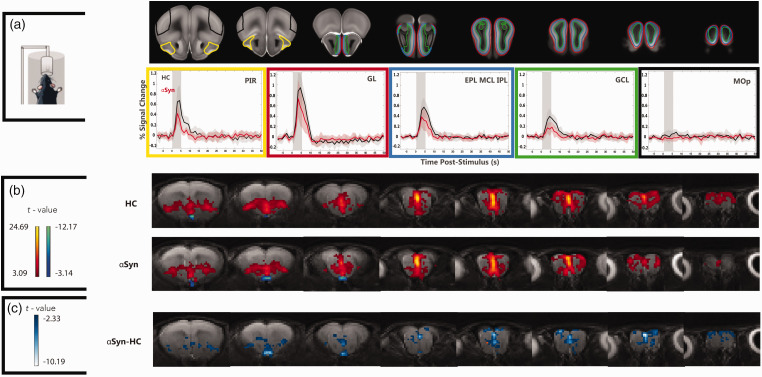
Olfactory fMRI in human α Syn tg mice reveals decreased activation compared to healthy controls. (a) ROI analyses along the olfactory pathway. BOLD activity was detected in all olfactory ROIs (the signals shown were averaged along all epochs and mice) in both groups, while an unrelated area (MOp) showed no activity upon olfactory stimulation, as expected. The vertical gray shaded area is the stimulation period. Red/black lines and shaded areas represent mean signal and 95% CI in the ROIs for α Syn and HC, respectively. Signals from PIR, GL, EPL_MCL_IPL and GCL are lower in the α Syn group (red traces) compared to the control group (black traces) and (b) BOLD-fMRI mapping in the olfactory pathway of α Syn and HC mice. Voxelwise activation maps for each group, obtained from *N = *10 HC and *N = *10 α Syn mice, reveals strong activation in the HCs along the entire pathway. In the α Syn group, BOLD responses are weaker, and lower overall *t*-values are observed, especially in more medial aspects of the bulb and (c) quantification of BOLD difference between the groups shows weaker activity along multiple olfactory pathway areas, including PIR, GL, EPL_MCL_IPL and GCL, at the *p* < 0.01 level.

Raw data from the fMRI EPI experiments in both pathways exhibited little susceptibility-induced distortions or motion artifacts (Video S1). tSNR maps showed a high tSNR of >30 in the relevant visual and olfactory areas (c.f. Figure S1 for tSNR maps from representative mice). Figure 2(a) presents BOLD-fMRI signals in ROIs placed along the olfactory pathway. Responses in the healthy control group were robustly observed, peaking ∼3 seconds after stimulus onset and decaying back to baseline around 10–15 seconds after the peak. The stimuli were stronger in the more external layers and weaker in the internal layers of the MOB. Note that in the control area, no such responses were observed, as expected. In the α Syn group, BOLD-fMRI responses were also noted along the olfactory pathway (and not in the control area), but with qualitatively weaker amplitude compared to HC’s. The largest decreases compared to HC were noted in the GCL and PIR.

Given the robust BOLD fMRI responses along the pathway, we turned to a more quantitative voxelwise analysis (Figure 2(b)). Upon olfactory stimulation, healthy controls exhibited robust activation in the entire olfactory pathway, reaching t-values of up to ∼23. The activation patterns of α Syn mice appeared spatially similar, with a maximum t-value reaching ∼25, but were generally of lower intensity compared with HCs. To more directly quantify the differences between the two groups, we performed group comparisons for the difference at a threshold of *p* < 0.01 (Figure 2(c)). Note the clearly lower activation in α Syn mice when compared to HC, especially around the midline and more medial areas of the system (Figure 2(c)).

### BOLD-fMRI signals in the visual pathway of α Syn mice are also disrupted

**Figure 3.**
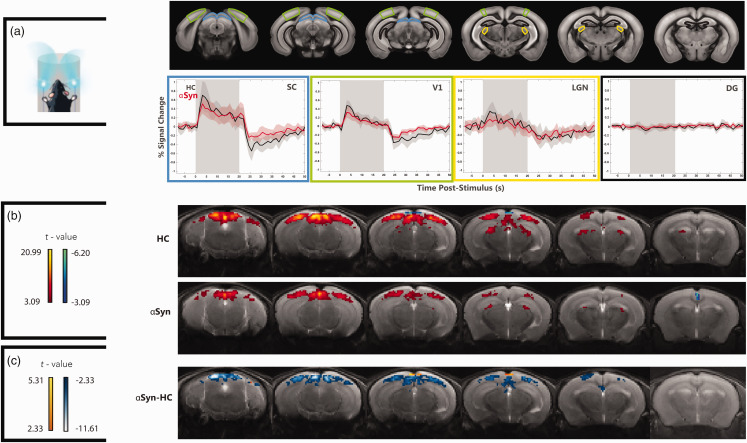
Visual fMRI in α Syn mice reveals decreased activation compared to healthy controls. (a) ROI analyses along the visual pathway. ROI analysis in the visual pathway and a control area, reflecting mean BOLD activity (averaging all epochs in the paradigm) for the two groups. The vertical gray shaded area is the stimulation period. Red/black lines and shaded areas represent mean signal and 95% CI in the ROIs for α Syn and HC obtained from all animals, respectively. (b) BOLD-fMRI group maps (HC vs α Syn) in the visual pathway, averaged across the *N = *13 HC mice and *N = *12 α Syn mice. In the α Syn group, BOLD responses are weaker, and lower overall *t*-values are observed in all analyzed areas and (c) Quantification of BOLD difference between the groups shows weaker activity along multiple visual pathway areas, including SC and V1, at the *p* < 0.01 level.

To investigate whether sensory disruptions extend to other sensory modalities, we performed binocular visual stimulation experiments (Figure 3). The fMRI signals in ROIs placed along the visual pathway (Figure 3(a)) show robust signals in all cortical and subcortical brain areas, while unrelated control areas (DG) exhibit no signals, as expected. A strong post-stimulus undershoot can be observed in SC and V1 for both groups. In the α Syn group, the BOLD response magnitude was qualitatively weaker than HC counterparts, especially in the SC and V1, also evidencing slightly different (less pronounced) post-stimulus undershoots. In the LGN, such differences appeared marginal.

We then quantified the voxelwise responses in both groups (Figure 3(b)). In HCs, the visual stimulation elicited clear activity along the visual pathway, while in the α Syn group, lower t-values were observed in most areas. The group difference maps calculated for *p* < 0.01 clearly highlight the activation deficits in the α Syn, mainly in SC and V1 (Figure 3(c)). Interestingly, LGN activity did not pass the statistically significant threshold of voxelwise differences between the groups.

### c-FOS levels reveal neural contributions to the weaker fMRI signals in α Syn mice

**Figure 4.**
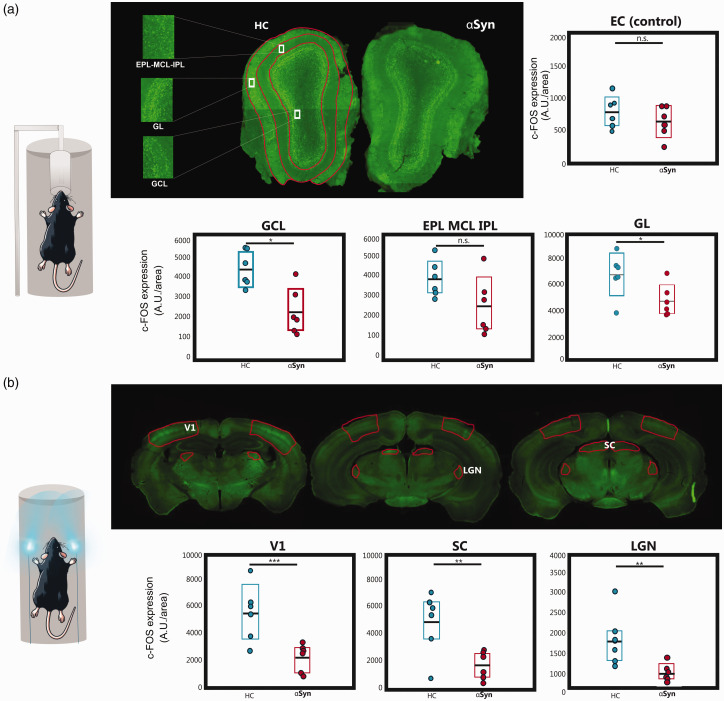
c-FOS levels. (a) Histological slices of the c-FOS levels in the MOB, and the analyzed ROIs (highlighted in red). Good expression levels were observed for both groups. ROIs were chosen anatomically (when needed, encompassing multiple slices for quantification) for *N = *6 animals per group. Analysis of c-FOS protein levels in each ROI using a *t*-test (*p < 0.05, **p < 0.01, and ***p < 0.005). Colored bars indicate the 95% CI and the black horizontal line the mean. Statistically significant reductions in c-FOS levels after olfactory stimulation were observed in GL and GCL but not in the EPL_MCL_IPL and in the control (unrelated) area, the entorhinal cortex (EC). Effect size estimates were *d = *1.449 in the GCL, *d = *1.265 in the EPL_MCL_IPL and d = 1.3661 in the GL, indicating a very large effect size; and *d = *0.623 in the EC (medium effect size) and (b) Same as (a) but for relevant slices of the visual pathway. Statistically significant reductions in c-FOS levels after visual stimulation were observed for the α Syn group compared to its HC littermate in the areas of the visual pathway. Cohen’s *d* values were: *d = *1.975 in the V1; *d = *1.665 in the LGN; *d = *1.784 in the SC, reflecting very large effect sizes.

Given the complexity of neurovascular couplings,^78^ we probed the neural contributions to the observed fMRI deficits using c-FOS experiments (Figure 4(a)). Microscopy in a representative slice of the MOB (HC (left) and α Syn (right)) reveals robust c-FOS staining in both groups following olfactory stimulation. When quantified, c-FOS levels were lower in the MOB of the α Syn group compared to the HC. In the GL expression levels decreased by 28% between the two groups while in the GCL they decreased by ∼50%, both with *p* < 0.05. In EPL_MCL_IPL, a decreasing trend was noted, although not reaching statistically significant levels. Nevertheless, the effect size estimated for this region was *d = *1.265, indicating a very large effect size. As expected, no baseline difference in c-FOS levels were found in a control, olfactory-unrelated area.

We then performed the same experiments along the visual pathway, following visual stimulation (Figure 4(b)). Robust c-FOS staining along the visual pathway is clearly observed. When quantified, c-FOS levels in the αSyn group were significantly reduced when compared to the HC in V1, SC, and LGN areas by up to ∼70% (*p* < 0.01), while a control region showed, as expected, no statistically significant differences between the groups.

### CBF measurements reveal vascular deficits in α Syn mice

**Figure 5.**
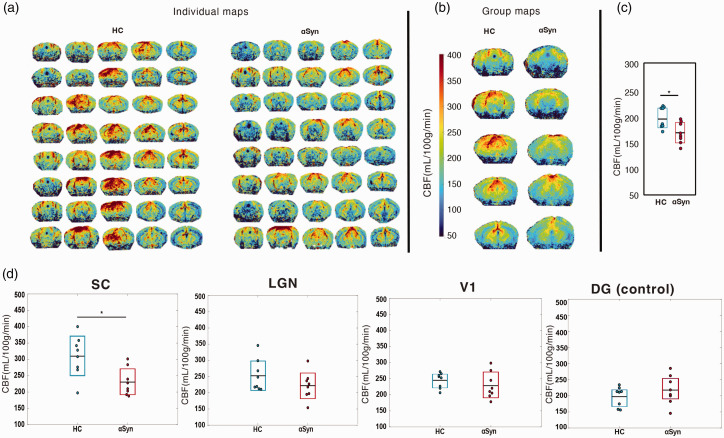
pCASL CBF maps. (a) Quantitative CBF maps for *N = *8 HC and *N = *8 α Syn individual animals (5 slices each). (b) Average group maps of the animals shown in (a). (c) CBF quantification. Quantification of brain perfusion of the studied groups. Bars indicate the 95% CI and the black horizontal line the mean. Statistically significant differences were found (*t*-test, *p* < 0.05) between groups, with *d = *1.409 (very large effect size) and HC animals exhibiting higher CBF compared with the α Syn group and (d) ROI analysis. Statistically significant reduction of CBF was found in the SC (*t*-test, *p* < 0.05) but not in the other areas, including in the control ROI. Cohen’s *d* values were: *d = *0.5087 in DG (control); *d = *0.4832 in the V1; *d = *0.6844 in the LGN; and *d = *1.4840 in the SC, reflecting medium to very large effect sizes.

To probe potential vascular effects, we mapped brain-wide CBF using pCASL measurements (Figure 5). Individual maps are shown in Figure 5(a), revealing relatively good agreement between different animals of each group. Upon averaging the individual CBF maps, the group maps clearly demonstrated lower perfusion in the αSyn group (Figure 5(b)). When quantified, the brain-wide CBF was ∼10% lower in the α Syn group compared to HC’s (*p* < 0.05) (Figure 5(c)). Finally, the ROI analysis (Figure 5(d)) showed statistically significant reduction of CBF in the SC but not in the other areas, nevertheless following the same trend, and in the control ROI (DG). Cohen’s d values were: d = 0.4832 in the V1; d = 0.6844 in the LGN; and d = 1.4840 in the SC, reflecting medium to very large effect sizes.

## Discussion

Dopaminergic dysfunction and death, alongside with *α*Syn aggregation in the brain,^79^ are established pathological alterations in PD leading to progressive motor symptoms and cognitive decline.^15,80^ Interestingly, olfactory and visual sensory deficits have been reported in PD patients, in some cases many years before the motor symptoms onset.^16,18^ Other sensory effects include increases in tactile and thermal sensitivity, decreases in mechanical pain perception, and reduction in epidermal nerve fibers.^81^ PET and fMRI experiments suggest that lower brain metabolic activity in sensory areas,^82^ but it has been difficult to pinpoint whether signaling deficiencies,^24^ damage in sensory areas,^83^ or associated changes in perfusion contribute to these effects.^84^

Using a well-established human *α*Syn transgenic mouse model of PD-like Synucleinopathy,^29,58,60^ we assessed evoked activity in the brain’s sensory networks. Our multimodal approach included fMRI for interrogating entire pathways as well as c-FOS expression and CBF mapping for probing the neural and vascular components of fMRI signals, respectively, thereby providing an integrated view from a neurovascular perspective. Weaker fMRI signals were found in both olfactory and visual sensory networks in the α Syn group, and the c-FOS and CBF experiments validated that decreased neural activity in these networks, combined with reduced blood flow rates, underpin the weaker fMRI signals. To our knowledge, this is the first observation of a combined visual and olfactory sensory aberration in the brain activity of PD rodent models in general and the α Syn model in particular. This provides an opportunity for future studies to interrogate how sensory deficits progress along the disease (and perhaps lead to early imaging biomarkers), as well as to probe the dopaminergic impact on these systems e.g. with optogenetics^85^ or pharmacological^86^ interventions, combined with visual^64,87^ and/or olfactory^88,89^ stimulation in the MRI scanner, as well as with resting-state studies.^90^ Further future experiments could correlate α Syn expression in these areas with aberrant activity.^8^

Most of the previous reports investigating the biological underpinnings in animal models of PD have mainly focused on the olfactory system. In an injected α Syn mouse model of PD, aggregations were found to occur progressively from the bulb to the piriform cortex,^91^ while others described reduced neurogenesis along the entire olfactory pathway^29^ and behavioral^92^ experiments indicating olfactory system deficits.^93^ Our results are aligned with these studies, as we also found decreased fMRI signals in the olfactory pathway from the bulb to the piriform cortex. Another recent study^93^ selectively expressing α Syn in the bulb found a decrease of granule cell activity in the GCL, consistent with our fMRI as well as our c-FOS expression experiments, but also reported increased activity in mitral and tufted cells in the respective layers. Our findings, however, indicate that the decrease in activity is shared among all olfactory areas. The discrepancy could arise from either the different models used, from different α Syn expression patterns, from population-level vs. single cell readouts, and/or from disruptions in neurovascular coupling that can make it more difficult to ascribe decreased fMRI signals to decreased input/output to/from an area,^94^ or directly relate the findings to changes in excitation/inhibition balances.^39^ Our current fMRI work suggests that all MOB layers are affected (as well as higher areas, e.g. PIR), providing a target for future studies in the olfactory network. The significant reductions in c-FOS expression in both the GL and GCL of αSyn mice suggests that αSyn pathology disrupts both sensory input and inhibitory feedback mechanisms in the olfactory bulb, aligning with prior evidence indicating that olfactory deficits are an early hallmark of PD. While the EPL_MCL_IPL did not reach statistically significant levels, a trend was observed and future studies with larger sample sizes may help clarify their role in PD-related dysfunction. The observed discrepancies between BOLD fMRI signals and c-FOS expression may be attributed to the global nature of hemodynamic responses rather than purely localized neuronal activity. Previous research has demonstrated that BOLD signals integrate vascular responses from multiple regions, often blurring layer-specific activity.^43^ In addition, unfortunately, our histological analyses were limited to the MOB, and PIR was not examined in this study, making a direct comparison between fMRI and c-FOS in that area impossible. Future studies integrating broader histological analyses and more complex fMRI paradigms can provide mutually-reinforcing insights into the dysfunction in the model. One potentially interesting avenue includes, for instance, testing degree of habituation: it has been previously shown that repeated exposure to specific odors may cause olfactory habituation and concomitant attenuation of fMRI signals.^95^ Although the experimental paradigm used in our study was not suitable for such a measurement,^96,97^ future studies could target habituation using longer odor exposures and more stimulation repetitions to provide additional insights into olfactory processing deficits in this PD model.

In this study, we aimed to go beyond a single sensory system to uncover a potentially more generalized sensory dysfunction in the brain. Although visual symptoms are reported in PD patients,^18^ they have been rather rarely investigated in the context of mouse models, especially at a network-level perspective. In a 6-OHDA rat model of dopaminergic cell loss^98^ (without α Syn deposition), signals in SC – a major input junction of the visual pathway – were found to be enhanced, suggesting that dopaminergic dysfunction alone could increase activity (consistent e.g. with hallucinations). However, our results suggest decreased activity in SC and other visual areas in the α Syn model brain – both from the fMRI and the c-FOS perspectives. Furthermore, a recent study recording in the SC and comparing with fMRI signals found a nearly perfect correlation between multiunit activity (MUA) and fMRI signals in the SC in rats,^64^ suggesting that reduced fMRI signals in this area indicate reduced neural activity (as also consistent with our c-FOS data). Despite the difference in species and some potential vascular effects (as measured with our CBF experiments and consistent with another recent study^48^ indicating reduced blood flow in the same model), it is likely that our results predominantly reflect decreased neural activity in the SC upon simple visual stimulation. This could suggest an interesting opposing effect of α Syn and dopaminergic dysfunction, i.e., that dopaminergic dysfunction acts to increase neural signals, while α Syn expression reduces activity. However, we cannot deconfound a potentially decreased retinal input into the visual pathway due to retinal and/or optic nerve α Syn aggregation,^99^ and the interaction between all these factors remains to be investigated in future studies. It is also interesting to note that while c-FOS expression was significantly reduced in the LGN area in *α*Syn mice compared to the HC, no significant differences in BOLD fMRI signals were detected. This divergence could reflect the inherent differences between c-FOS immunohistochemistry and fMRI in assessing neuronal activity: while c-FOS detects spiking activity and transcriptional activation following synaptic input with high sensitivity, BOLD fMRI signals are more indirect^40^ and reflect the neurovascular coupling, with potentially lower sensitivity in lower SNR regions^100^ such as LGN.^101^ The relatively small size of the LGN further increases the likelihood of partial volume effects, potentially masking localized BOLD signal variations. Furthermore, in PD and related *α*-synucleinopathies, growing evidence suggests that neurovascular coupling is impaired, potentially making BOLD fMRI less sensitive to these changes.^102^ In our study, SC exhibited the only statistically significant decrease in CBF while V1 and LGN remain unaffected. This aligns with previous reports of impaired neurovascular function in PD models, where regional-specific differences in blood flow may contribute to altered sensory processing and metabolic dysfunction. SC is critically involved in visual attention and sensorimotor integration, and disruptions in its vascular supply may contribute to deficits observed in PD. In the future, higher-quality CBF mapping, along with hypoxia/hyperoxia challenges or measurements of CBF stability over time could better assess vascular reactivity and assist in further decoupling neural from vascular effects. Still, our fMRI results point to a massive disruption of brain activity at the visual network level, involving mainly the extrageniculate pathway and cortex. Previous studies have found disruptions in resting-state networks both in patients^103^ and in the animal models^104^ such as mice and rats, consistent with the network-level findings in our current work.

Our findings indicate decreased neural activity coupled with decreased CBF upon evoked activity in the α Syn model. It is interesting to note that while c-FOS levels were reduced by more than a factor of two in the visual pathway regions and at least the GCL in the olfactory pathway, the CBF measurements indicated only a ∼10% decrease in flow rate. While our work does not offer the possibility to directly relate these metrics to BOLD-fMRI signals, and previous evidence indeed indicates an effect of α Syn aggregation on vasculature,^105^ we nevertheless propose that it is plausible that the decreased fMRI signals observed here were more strongly influenced by the decreased evoked activity (reduced c-FOS expression) than damage to the blood vessels (reduced CBF levels). Future experiments could interrogate the neurovascular unit and its reactivity using gas challenges^106^ and more quantitative modeling of BOLD signals^107^ to better understand neurovascular coupling in such models.

Finally, we note in passing that in this study, significant differences at the whole brain volume level (Figure S2) were not observed here, while previous studies demonstrated that brain volume alterations in PD models can occur in region-specific and model-dependent ways.^108^ The absence of whole-brain volume loss in our study aligns with findings in the 6-OHDA model, where no significant basal ganglia atrophy was observed.^109^ Future investigations should explore region-specific volumetric analyses to determine whether αSyn accumulation preferentially affects certain brain structures before global atrophy becomes evident.

## Limitations

As in every study, we identify several limitations of our work. First, we did not quantify α Syn levels in the brains of each individual animal used in the study. Therefore, it is not possible to directly link α Syn levels to network level activity deficits. Previous studies^60,110^ in this mouse line have shown that it does not exhibit α Syn aggregates *per se*, but rather overexpresses the protein across the entire brain. In other α Syn mouse models, α Syn is expressed in various neuronal populations and areas, including in the midbrain, cerebellum, brainstem, and spinal cord.^93^ However, the expression patterns are expected to be heterogeneous and likely are more prominent in the dopaminergic regions, slowly spreading out to other brain areas.^111^ This heterogeneous pattern can be useful in future studies attempting to directly correlate α Syn load with network-level sensory (or other) aberrations.

In addition, we cannot deconvolve whether the effects shown here originate from local circuit dysfunction, aberrant inputs from sensory organs, and/or more global dopaminergic (and/or other secondary modulatory effects) dysfunction^112^ and its downstream impact. Other factors, such as mitochondrial dysfunction,^113^ 3,4-dihydroxyphenylacetaldehyde (DOPAL) toxicity,^114^ and brain atrophy^49^ can also contribute to sensory dysfunction. Tackling these questions will require more specific experiments, involving e.g. optogenetics, lesion studies, and invasive recordings. Still, previous experiments lesioning dopaminergic areas typically show a “disinhibiting” effect,^115^ thereby resulting in enhanced activity at least in some cell types. The effects in this study were opposite, suggesting more local and/or input aberrations as the main contributors to the decreased activity observed.

From the experimental perspective, it is worth mentioning that the weight of our healthy controls at 9 months old was significantly higher than the α Syn mice. While this could confound the interpretation of our fMRI results in the sense that neurovascular coupling (as well as response to anesthetics) may be influenced by obesity,^116^ we could not find any significant correlation between response amplitude and animal weight. Given that fMRI activity was clearly detected in these mice and that, if anything, obesity would likely decrease fMRI signals,^117^ we posit that the difference between α Syn and HC cannot be fully explained by weight differences. In future studies, this effect may be further assessed by restricting the diet of the HCs such that their weight is more controlled, although that introduces confounds of its own. Another alternative would be to perform experiments in weight-matched animals but this would require mice of different ages, which likely would make disease progression a significant confounding factor. We further note that we did not perform behavioral experiments: future studies could correlate behavioral readouts with network level activity to establish the relevant links.

In the context of CBF mapping, we note that olfactory ROIs were not included in our pCASL experiments due to our initial study design, which prioritized whole-brain coverage with a focus on the visual system. Additionally, technical challenges in acquiring high-quality whole-brain pCASL images made it difficult to reliably assess CBF in the olfactory bulb. Future studies should aim to target more specifically the olfactory regions to further explore potential perfusion deficits in sensory systems commonly affected in PD.

Finally, we used only male mice for the PD group in this study, since in this PD genetic model, the males are hemizygous,^60^ always expressing the PD phenotype while females only carry the mutation. This approach prevents confounding effects related to the absence of *α*Syn overexpression in female carriers, ensuring a more accurate assessment of the disease-related effects but may entail a bias towards male traits. While sex differences in PD models have been reported,^118^ our study design aimed to minimize additional variability by maintaining a homogeneous sample. Nonetheless, future studies incorporating both male and female cohorts, using alternative genetic strategies, could provide further insights into potential sex-specific differences.

## Conclusions

Our study reveals broad, network-level sensory deficits in fMRI signals upon evoked activity in the olfactory and visual pathways, occurring in tandem at 9 months of age in a human α Syn tg mouse of PD. These deficits were shown to have neural origins as well as vascular contributions, supporting the notion of decreased sensory activity in the brain given a stimulus. Our results open novel perspectives for future investigations of sensory deficits during the progression of PD and other Synucleinopathies, as well as for the development of early biomarkers for PD.

## Supplemental Material

sj-pdf-1-jcb-10.1177_0271678X251338952 - Supplemental material for Neural and vascular contributions to sensory impairments in a human alpha-synuclein transgenic mouse model of Parkinson’s disease Supplemental material, sj-pdf-1-jcb-10.1177_0271678X251338952 for Neural and vascular contributions to sensory impairments in a human alpha-synuclein transgenic mouse model of Parkinson’s disease by Ruxanda Lungu, Francisca F Fernandes, Sara Pires Monteiro, Tiago F Outeiro and Noam Shemesh in Journal of Cerebral Blood Flow & Metabolism

sj-pdf-2-jcb-10.1177_0271678X251338952 - Supplemental material for Neural and vascular contributions to sensory impairments in a human alpha-synuclein transgenic mouse model of Parkinson’s disease Supplemental material, sj-pdf-2-jcb-10.1177_0271678X251338952 for Neural and vascular contributions to sensory impairments in a human alpha-synuclein transgenic mouse model of Parkinson’s disease by Ruxanda Lungu, Francisca F Fernandes, Sara Pires Monteiro, Tiago F Outeiro and Noam Shemesh in Journal of Cerebral Blood Flow & Metabolism

sj-mp4-3-jcb-10.1177_0271678X251338952 - Supplemental material for Neural and vascular contributions to sensory impairments in a human alpha-synuclein transgenic mouse model of Parkinson’s disease Supplemental material, sj-mp4-3-jcb-10.1177_0271678X251338952 for Neural and vascular contributions to sensory impairments in a human alpha-synuclein transgenic mouse model of Parkinson’s disease by Ruxanda Lungu, Francisca F Fernandes, Sara Pires Monteiro, Tiago F Outeiro and Noam Shemesh in Journal of Cerebral Blood Flow & Metabolism

## Data Availability

The authors confirm that the data supporting the findings of this study will be made available on a free repository. The code used to analyze the fMRI data analysis will be shared upon request.
