# Supplementary figures and images for: Neural and vascular contributions to sensory impairments in a human alpha-synuclein transgenic mouse model of Parkinson’s disease
Source: J Cereb Blood Flow Metab. 2025 May 7;45(9):1654–69. doi: 10.1177/0271678X251338952 (PMC12058711; doi:10.1177/0271678X251338952)

### A) Olfactory areas

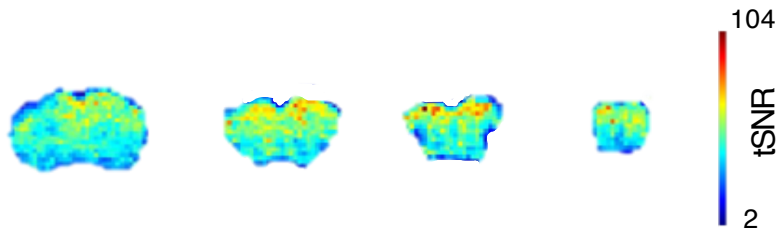

### B) Visual areas

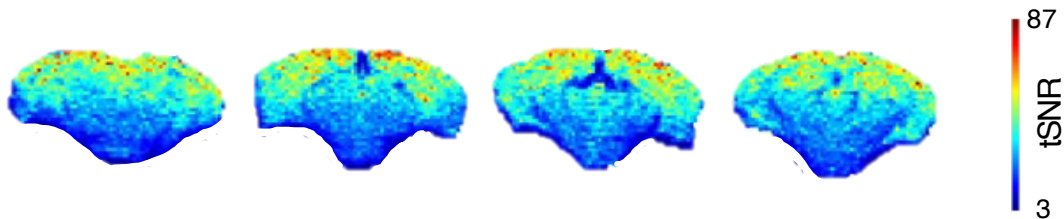

Supplement: sj-pdf-1-jcb-10.1177_0271678X251338952 - Supplemental material for Neural and vascular contributions to sensory impairments in a human alpha-synuclein transgenic mouse model of Parkinson’s disease [file sj-pdf-1-jcb-10.1177_0271678X251338952.pdf]

A)

HC

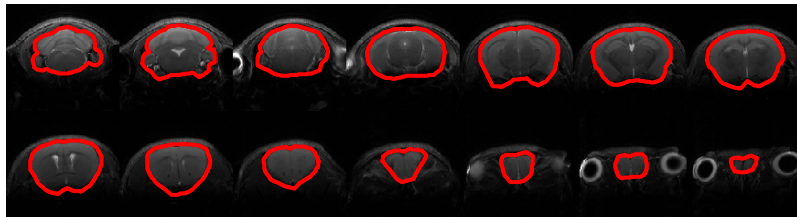

aSyn

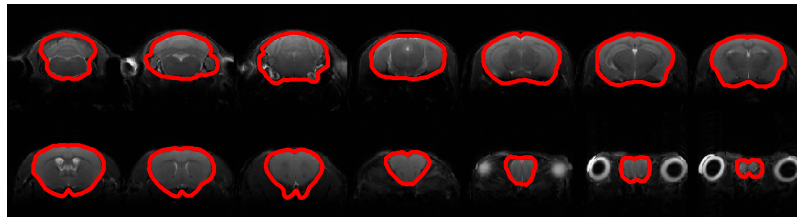

B)

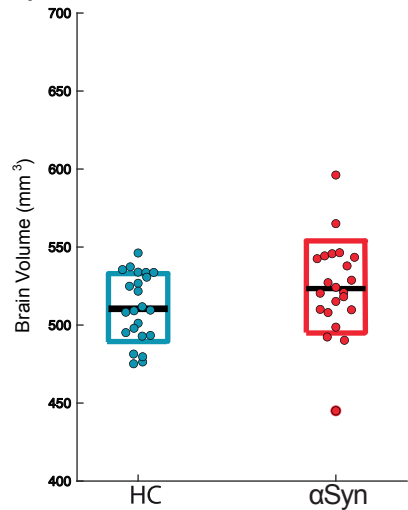

Supplement: sj-pdf-2-jcb-10.1177_0271678X251338952 - Supplemental material for Neural and vascular contributions to sensory impairments in a human alpha-synuclein transgenic mouse model of Parkinson’s disease [file sj-pdf-2-jcb-10.1177_0271678X251338952.pdf]
